# Effects of dietary supplementation of Vitamins E and C on oxidative stress induced by a Nigerian velogenic strain of the Newcastle disease virus (KUDU 113) in the brain and bursa of Fabricius of broiler chickens

**DOI:** 10.14202/vetworld.2021.2452-2461

**Published:** 2021-09-21

**Authors:** Obianuju Nkiruka Okoroafor, Temitope Mofoluso Ogunniran, Nkechi Harriet Ikenna-Ezeh, Ikechukwu John Udeani, Jacinta Ngozi Omeke, Wilfred Sunday Ezema, Boniface Anene

**Affiliations:** 1 Department of Veterinary Medicine, Faculty of Veterinary Medicine, University of Nigeria, Nsukka, Nigeria; 2 Department of Veterinary Pathology and Microbiology, Faculty of Veterinary Medicine, University of Nigeria, Nsukka, Nigeria; 3 Veterinary Teaching Hospital, Faculty of Veterinary Medicine, University of Nigeria, Nsukka, Nigeria.

**Keywords:** hematologic parameters, Newcastle disease, oxidative stress, Vitamin C, Vitamin E antibody titer

## Abstract

**Background and Aim:**

Newcastle disease (ND) is widely recognized as an extremely harmful and contagious disease of birds. Therefore, the present study aims to evaluate the effect of oxidative stress induced by the virulent ND virus (NDV) (KUDU 113) on the plasma, brain, bursa of Fabricius, NDV antibody response, and hematology as well as the ameliorative effect of the individual or combined use of Vitamins E and C on the clinical signs of NDV-infected chickens.

**Materials and Methods:**

In this study, a total of 150 broiler chickens were included and divided into five groups: Group 1, nonsupplemented and unchallenged chickens (UCC); Group 2, nonsupplemented and challenged chickens (ICC); Group 3, Vitamin C-supplemented + challenged chickens; Group 4, Vitamin E-supplemented + challenged chickens; and Group 5, Vitamins E and C-supplemented + challenged chickens. Groups 3, 4, and 5 were supplemented with Vitamins E and C (33 and 400 mg/kg/day, respectively). Virus challenge was done with 0.1 ml of KUDU 113 7 days after the start of vitamin inclusion in their diet. Concentrations of glutathione (GSH), malondialdehyde (MDA), nitric oxide (NO), superoxide dismutase (SOD), and catalase (CAT) were analyzed in the plasma, brain, and bursa on days 0, 3, and 7 post-infection (pi) using the biochemical method. The blood samples were randomly collected from five chickens in each group for antibody response and hematological analyses on day 0 previtamin treatment and at 0, 3, 7, 10, 14, and 21 days pi (dpi).

**Results:**

A significant increase in the concentrations of MDA and NO in the NDV-challenged chickens was observed when compared with the UCCs. Moreover, a significant decrease in GSH concentration was observed in the NDV-challenged chickens when compared with the UCCs. The activities of CAT and SOD were reduced markedly in the NDV-challenged chickens. Increases in the mean antibody titers were observed in the NDV-challenged group when compared with the UCCs from days 3 to 21 pi. The mortality rates of groups 1, 2, 3, 4, and 5 were 0%, 30%, 3.3%, 3.3%, and 26.6%, respectively.

**Conclusion:**

The findings of this study suggest that KUDU 113 causes oxidative stress in the brain and bursa of Fabricius of chickens. Individual supplementation with Vitamin E or C was found to be more effective in ameliorating oxidative stress, improving the immune response, and reducing mortality in KUDU 113 infections than the combined supplementation of Vitamins C and E.

## Introduction

Newcastle disease (ND) is a dangerous viral poultry disease characterized by pathologies in the digestive system, reproductive system, respiratory system, nervous tissues, and lymphoid tissues. The disease is attributed to numerous economic losses in the poultry industry throughout the world [1]. Although a significant improvement has been observed in the diagnosis and vaccination of poultry against ND, constant outbreaks are still recorded in both vaccinated and unvaccinated birds, and the molecular mechanism of the ND virus (NDV) must be further investigated [2]. The NDV is an avian orthoavulavirus that belongs to the genus *orthoavulavirus* in the subfamily Avulavirus and family Paramyxoviridae [3]. The NDV is categorized on the basis of pathogenicity as asymptomatic, lentogenic mesogenic, and velogenic strains of the virus [4]. The mesogenic and velogenic strains are responsible for the heavy mortality and systemic infection observed in poultry. The NDV is also classified as viscerotropic, pneumotropic, and neurotropic strains on the basis of the virus affinity for nervous and visceral organs [5]. The viscerotropic strain of ND causes hemorrhagic lesions in the intestine, thymus, and bursa of Fabricius, whereas the neurotropic strain causes neurolytic signs, such as paresis and paralysis along with respiratory signs [5-7]. The by-products of metabolic processes in the body involve the production of a wide range of reactive substances. These substances are biologically reactive oxygen species (ROS) and reactive nitrogen species (RNS). The ROS induces substances of high and low reactivity, such as hydroxyl radicals, superoxides, and low reactivity, and hydrogen peroxide, whereas the RNS substances include nitric oxide (NO) and hydrogen peroxide [8]. The production of these reactive substances, if regulated effectively, aids the cell signaling, the regulation of cytokines, neuromodulation, transcription, apoptosis, and transport [9]. These reactive substances also play key roles in the host-pathogen interaction, which induces the recognition of pathogens, activation of the host defense system, and gene expression. [10]. To reduce ROS- and RNS-induced alterations, organisms have definite antioxidant defense systems involving enzymatic and non-enzymatic components, such as catalase (CAT), superoxide dismutase (SOD), glutathione S-transferase (GST), glutathione (GSH), thioredoxin, melatonin, carotenoids, Vitamin E, and Vitamin C [11]. The ND virus is an RNA virus known to induce oxidative stress by increasing the production of reactive substances [12]. Recent studies have shown that mesogenic and velogenic NDV infections can cause oxidative stress in the brain by decreasing the activities of GST, SOD, and CAT [13,14] as well as in the bursa of Fabricius by increasing the macrophage population, NO levels, and malondialdehyde (MDA) content [15].

Vitamins E and C are well-known potent antioxidants. Vitamin E is actively involved in the development of the nervous system [16]. It is mostly found in cellular membranes and guards the cells against oxidative stress by scavenging reactive species. It may also be neuroprotective [17]. Vitamin E supplementation has been shown to improve immune function against viral pathogens and can modulate T cell function and cytokines [13,18]. Vitamin C plays a necessary role in various biosyntheses and is also important in regulating diverse reactions, such as the secretion of corticosteroids, regulation of body temperature, and activation of the immune system [19]. Vitamin E scavenges free radicals and compensates for the decrease in reduced GSH [18], whereas Vitamin C functions as a ROS scavenger and is effective in reducing oxidative damage by increasing the oxygen concentrations and apoptosis [15]. Vitamin E has been reported to ameliorate NDV-induced oxidative stress and alleviates tissue damage in the brains and intestines of chickens [2,14]. This shows that the dietary supplementation of antioxidants may be a promising approach to reduce economic losses caused by virus-induced oxidative stress [20].

Therefore, this study aims to determine the effect of oxidative stress induced by a Nigerian velogenic strain of the NDV (KUDU 113) on the brain, bursa of Fabricius, and plasma of chickens as well as to evaluate the anti-oxidative role of Vitamins E and C individually or in combination and their ability to reduce mortalities associated with NDV infection.

## Materials and Methods

### Ethical approval

The animal protocol was approved (approval no. UNFVM/18/19/099) by the Faculty of Veterinary Medicine University of Nigeria Nsukka, Nigeria. Experimental animal ethics committee and in compliance with Principles of Laboratory Animal Care” [21].

### Study period and experimental design

The study was conducted from December 2019 to March 2020. Overall, 150-day-old broilers chickens were obtained from a commercial hatchery in Nigeria and housed in the Department of Veterinary Medicine experimental animal house. The experimental chickens were vaccinated with the Gumboro and LaSota vaccines on days 10 and 21, respectively. The vaccines were procured from the Vaccine Production Unit, National Veterinary Research Institute, Vom Jos (NVRI), Nigeria. They had access to feed and water ad libitum. Thereafter, the chickens were divided into five groups (30 in each group): Group 1, nonsupplemented and unchallenged chickens (UCC); Group 2, nonsupplemented and NDV-challenged chickens (ICC); Group 3, Vitamin C-supplemented (400 mg/kg) and NDV-challenged chickens; Group 4, Vitamin E-supplemented (33 mg/kg) and NDV-challenged chickens (Vitamin E-supplemented + challenged chickens); and Group 5, Vitamin E-supplemented (33 mg/kg/day) and Vitamin C-supplemented (400 mg/kg/day) and NDV-challenged chickens. Vitamins C and E were procured from Enhalor Biotechnology (Beijing, China) and the BASF group (Ludwigshafen, Germany), respectively. Vitamin supplementation was included in the daily feed diet of the broiler chickens on day 35. The velogenic NDV isolate, KUDU 113, originally isolated from ducks (duck/Nigeria/Plateau/Kudu/113/1991) [22] and was procured from NVRI, Vom, Plateau State, Nigeria. Groups 2 3, 4, and 5 were challenged with 0.1 ml of KUDU 113 containing 10^8.46^ 50% egg lethal dose (50) 7 days after the start of vitamin inclusion in their diet.

### Sample collection

Blood samples were collected from the jugular vein of five birds per treatment group at day 0 previtamin supplementation; 0, 3, 7, 10, 14, and 21 days post-infection (dpi) in heparin tubes and in plain tubes were immediately transferred to the laboratory. The blood samples in the heparin tubes were used to analyze hematological parameters, whereas the blood in plain tubes was allowed to clot and was serum harvested and stored at −80°C for the determination of antibody titers using the enzyme-linked immunosorbent assay, procured from Shenzhen Lvshiyuan Biotechnology Co., Ltd., China.

Three chickens per experimental treatment group were humanely killed via the intravenous injection of pentobarbital sodium (40 mg/kg) for the collection of cerebrum and bursa samples at 0, 3, and 7 dpi. The samples were put in separate microtubes and stored at −80°C until use.

### Preparation of tissue homogenates

The harvested tissue sections of the cerebrum and bursa of Fabricius were weighed and completely homogenized in phosphate buffer. The homogenates were centrifuged at 5000× *g* for 20 min. The resulting supernatant was used to assay for the oxidative stress indicators.

### Determination of MDA

MDA, an end product of lipid peroxidation, 0.5 mL of serum, or tissue homogenate was mixed with 20% trichloroacetic acid (TCA) (1:1) and incubated at room temperature. The samples were centrifuged at 2500× *g* for 10 min. Moreover, 1.0 mL of 1% thiobarbituric acid was added to the supernatant and the samples were placed in a boiling water bath (100°C) for 15 min. The contents were cooled on ice and centrifuged for 15 min at 2500× *g*. The absorbance (A) of the supernatant was read at 532 nm against a reagent blank using a spectrophotometer (Jenway 6305; Jenway, Essex, UK) [23].

### Determination of GSH

Reduced GSH in the serum and tissue was determined according to the method described by [24]; 0.5 mL of serum/tissue homogenate was mixed with 0.1 mL of 25% TCA and kept on ice for a few minutes. This was then centrifuged at 3000 g for 10 min, and 0.3 mL of the supernatant was mixed with 0.7 mL of 0.2 M sodium phosphate buffer (pH 8) and 2 mL of 0.6 mM Dithionitrobenezene. After 10 min, the yellow color obtained was measured at 412 nm using a spectrophotometer (Jenway 6305; Jenway, Essex, UK) against a reagent blank.

### Determination of NO

Total NO was determined based on the application of the Griess reaction following the conversion of nitrate to nitrite by metallic cadmium [25]. Two hundred and fifty microliters of serum or tissue homogenate was added to 700 mL of distilled water and then mixed vigorously with 50 mL of deproteinization solution (30% zinc sulfate). The mixture was incubated at room temperature for 15 min followed by centrifugation at 3000× *g* for 5 min. The deproteinized supernatant was transferred to a tube containing two pellets of washed cadmium beads and then incubated overnight at room temperature with agitation. After incubation, the sample was transferred to a tube and centrifuged for 5 min at 3000× *g*. Moreover, 300 mL of the sample was mixed briefly with 50 mL of color reagent 1 (sulfanilamide (p-aminobenzenesulfonamide) in 3N HCl), followed by the addition of 50 mL color reagent 2 (N-(1-naphthyl) ethylenediamine dihydrochloride in water) and mixed for 5 min at room temperature. The absorbance of the sample was then measured at 540 nm using a spectrophotometer (Jenway 6305; Jenway, Essex, UK) against a reagent blank.

### Determination of SOD

SOD activity was determined according to the method developed by Misra and Fridovich [26]; 0.5 mL of serum or tissue homogenate was diluted with an equal volume (0.5 mL) of distilled water, followed by the addition of 0.25 mL of ice-cold ethanol and 0.15 mL of ice-cold chloroform. This was thoroughly mixed using a cyclo-mixer and then centrifuged at 2500 rpm for 10 min. The supernatant was mixed with 1.5 mL of carbonate buffer (0.05M, pH 10.2) and 0.5 mL of 0.5 mM EDTA solution. The reaction was initiated by the addition of 0.4 mL of 3 mM epinephrine (Sigma, St. Louis, MO, USA), and the change in absorbance per minute was measured at 480 nm against a reagent blank.

### Determination of CAT

CAT activity was determined according to the method described previously by Sinha [27]; 0.04 ml of serum or tissue homogenate was added to 2.96 ml of H_2_O_2 (_0.2 M)-phosphate buffer (0.01 M, pH 7). From this mixture, 2 mL of dichromate/acetic acid reagent was used to stop the reaction with an interval of 1 min. The tubes were heated at 100°C for 10 min, cooled, and then centrifuged at 2500× *g* for 5 min to remove precipitated proteins. The changes in absorbance were recorded at 570 nm against the reagent blank using a spectrophotometer (Jenway 6305; Jenway, Essex, UK).

### Determination of NDV antibody titers

An enzyme-linked immunosorbent assay kit was used for the determination of antibody titers in the serum. The assay was performed according to the manufacturer’s instructions.

### Mortality rate

The experimental birds were observed twice daily for clinical signs of NDV infection post-challenge with the KUDU 113, and the mortalities were recorded.

### Hematology

Hematological analyses were performed immediately after collection. Packed cell volume (PCV) was determined using the microhematocrit method, whereas the hemoglobin concentration was determined using the cyanomethemoglobin method [28,29]. Red blood cell (RBC) and total white blood cell (WBC) counts were measured using the hemocytometer method. Erythrocytic indices were calculated using the standard formulas.

### Statistical analysis

The obtained data were subjected to a two-way analysis of variance using the GraphPad Prism version 5.2 for Windows (Graphpad Software, California, USA); variant means were separated using Bonferroni’s multiple comparison tests. The differences between means were considered significant at p<0.05.

## Results

### Changes in MDA concentration

**Figure-1:**
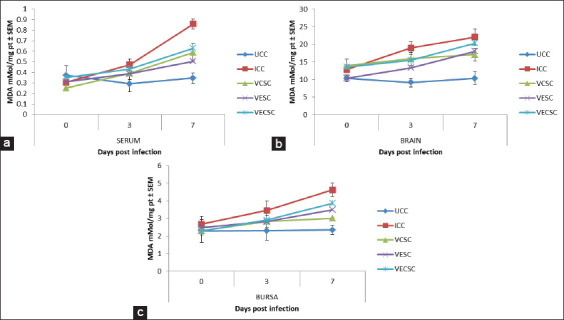
The concentration of malondialdehyde in the (a) serum, (b) brain, and (c) bursa of chickens challenged with Newcastle disease virus and supplemented with Vitamins E, C, and EC at 0-7 days post-infection.

The MDA concentrations in the serum, brain, and bursa were significantly (p<0.05) higher in the NDV-challenged group at 3 and 7 dpi when compared with the unchallenged group. Groups 2 and 5 had the highest increase in the MDA contents of the serum, brain, and bursa than the Vitamin C-supplemented or Vitamin E-supplemented groups (Figure-1).

### Changes in the NO concentration

**Figure-2:**
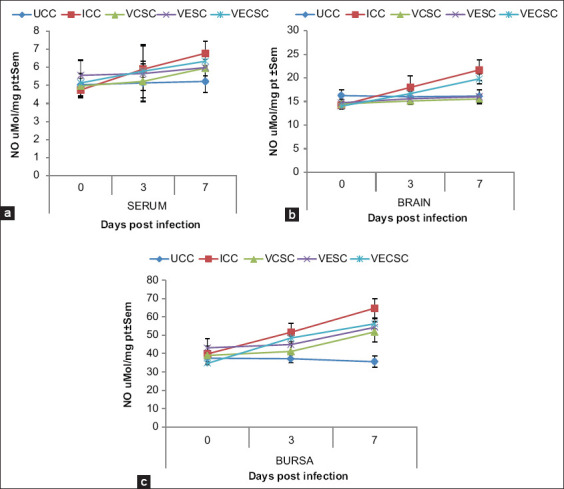
The concentration of nitric oxide in the (a) serum, (b) brain, and (c) bursa of chickens challenged with Newcastle disease virus and supplemented with Vitamins E, C, and EC at 0-7 days post-infection.

A significant (p<0.05) increase in NO concentration was observed in the serum, brain, and bursa of Fabricius of the NDV-challenged groups (Groups 2, 3, 4, and 5) at 3 and 7 dpi when compared with Group 1. However, the vitamin-supplemented groups had a lower NO concentration when compared with Group 2, whereas the NO contents in the serum, brain, and bursa of Fabricius of Group 5 were higher than those in Groups 3 and 4 (Figure-2).

### Changes in the GSH concentration

**Figure-3:**
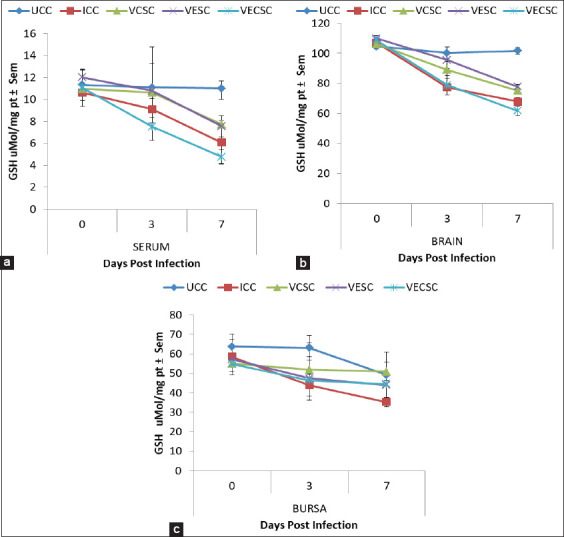
The concentration of glutathione in the (a) Serum, (b) Brain, and (c) Bursa of chickens challenged with Newcastle disease virus and supplemented with Vitamins E, C, and EC at 0-7 days post-infection.

No significant difference (p>0.05) was observed in the GSH concentrations in the serum, brain, and bursa of Fabricius of the NDV-challenged groups at 3 dpi (Figure-3). However, a marked decrease in GSH was observed in Group 2 compared with the groups supplemented with vitamins C, E, and E + C. The decrease in GSH content was more severe at 7 dpi than at 3 dpi. The decrease in GSH contents in the serum, brain, and bursa of Fabricius of Groups 3 and 4 was less than that of Groups 2 and 5.

### Changes in the SOD concentration

**Figure-4:**
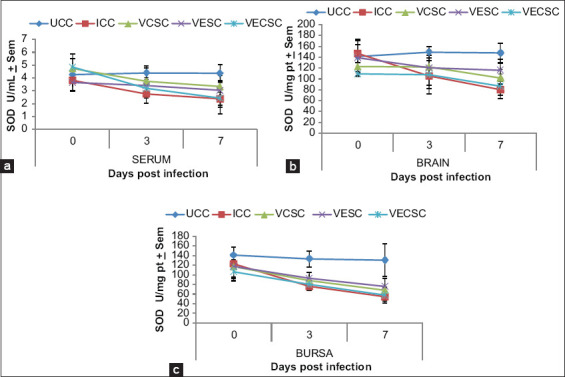
The concentration of Superoxide dismutase in the (a) serum, (b) brain, and (c) bursa of chickens challenged with Newcastle disease virus and supplemented with Vitamins E, C, and EC at 0-7 days post-infection.

The levels of SOD in the serum, brain, and bursa were not significantly (p>0.05) different in the NDV-challenged groups at 3 and 7 dpi (Figure-4). However, a decrease was observed in the SOD contents of the serum, brain, and bursa of Fabricius of the NDV-challenged/Vitamin E + C-supplemented chickens compared with the unchallenged group. The reduction was higher in Groups 2 and 5 than in Groups 3 and 4.

### Changes in the CAT concentration

**Figure-5:**
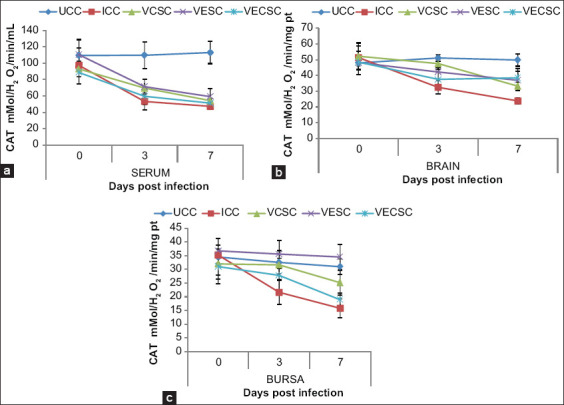
The concentration of catalase in the (a) serum, (b) brain, and (c) bursa of chickens challenged with Newcastle disease virus and supplemented with Vitamins E, C, and EC at 0-7 days post-infection.

No significant difference was observed between the concentrations of CAT in the serum, brain, and bursa of Fabricius of the NDV-challenged and supplemented/nonsupplemented chickens (p>0.05) and the UCC at 3 and 7 dpi (Figure-5). However, a reduction in the concentration of CAT in the serum, brain, and bursa of Fabricius of Groups 2, 3, 4, and 5 was observed. Furthermore, Groups 2 and 5 showed a higher reduction in CAT contents when compared with Groups 3 and 4.

### Mortality rate

**Figure-6:**
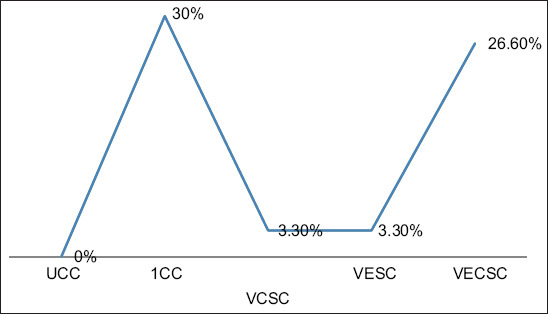
Mortality rate of chickens challenged with Newcastle disease virus and supplemented with Vitamins E, C, and EC.

Mortalities were first observed in Group 2 on day 5 pi and Group 5 on day 7 pi, whereas mortalities in Groups 3 and 4 were recorded on day 9 and 10 pi, respectively. The total mortalities were 0% for Group 1, 30% for Group 2, 3.3% for Group 3, 3.3% for Group 4, and 26.6% for Group 5 (Figure-6).

### Changes in the NDV antibody titers

**Figure-7:**
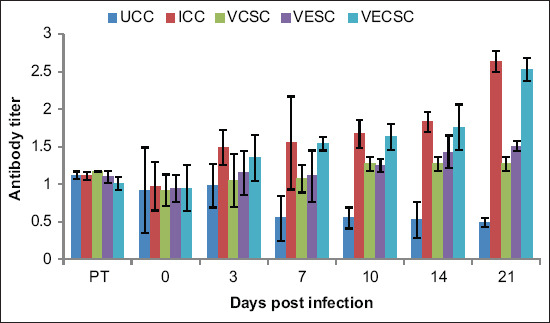
The antibody titer of chickens challenged with Newcastle disease virus and supplemented with Vitamins E, C, and EC.

No significant difference was observed in the ND hemagglutination inhibition titer of the NDV-challenged and Vitamin C-, Vitamin E-, and Vitamin E + C-supplemented groups. However, increases in antibody titers were observed in the NDV-challenged group from day 3 pi to day 21 pi. The antibody titer of Group 2 was higher than that of the vitamin-supplemented groups (Figure-7).

### Changes in hematology

**Figure-8:**
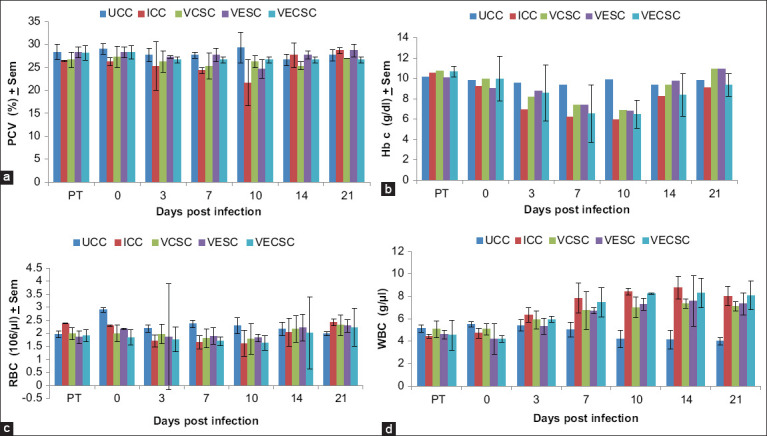
The packed cell volume (%), haemoglobin concentration (g/dl), red blood cell concentration (10^6^/µL) and white blood cell (g/µL) of chickens challenged with Newcastle disease virus and supplemented with Vitamins E, C and EC.

As shown in Figure-8, the Vitamins E, C, and E + C supplementation showed a nonsignificant effect (p>0.05) on the PCV, RBC, HB, and WBC of challenged birds when compared with the control group. However, reductions in the erythrocytic parameters and increases in the total WBC were observed in the groups challenged with KUDU 113 from day 3 pi to day 10 pi.

## Discussion

ND is attributed to severe economic losses in the poultry industry by damaging the nervous and lymphoid organs of chickens, even in vaccinated flocks. Okoroafor *et al*. [30] reported a mortality rate of 13.3% in vaccinated chicken flocks experimentally infected with the KUDU 113 strain of the NDV.

Studies have shown the replication of NDV in the brain of chickens [4,31], and the ability of NDV to replicate depends on the different isolates. Virulent ND depletes the bursa of Fabricius of chickens [15,30,32,33], and the severe pathology in the immune organ is associated with high levels of virus replication and intense inflammatory response [34]. Oxidative stress is due to the deregulation of cellular metabolism, resulting in the overproduction of ROS and RNS, which disturbs the cellular antioxidant enzymes and causes cellular damages [5]. The deleterious effect of oxidative stress is invalidated by some enzymatic (CAT and SOD) and non-enzymatic (GSH and Vitamins A, E, and C) antioxidant factors. Some researchers have reported that mesogenic and velogenic NDV cause oxidative stress in the brain, liver, heart, and bursa of Fabricius of chickens [2,13,15].

This study determined the effect of KUDU 113, a Nigerian velogenic NDV, on oxidative stress markers, antibody titers, mortality rate, and hematology in chickens supplemented or not supplemented with Vitamins E, C, and E + C.

MDA is an indicator of lipid peroxidation, which is an important factor in oxidative stress because free radicals are mainly produced in the lipid membrane and these free radicals can cause damage to the cell membrane, DNA, and proteins leading to death. In this study, a higher level of MDA was found in the serum, brain, and bursa of Fabricius of NDV-infected chickens, but less concentration was observed in the serum, brain, and bursa of Fabricius of chickens supplemented with Vitamins E and C. This finding is consistent with that of the previous studies on the brain [2], bursa [15], and intestinal mucosae [14], indicating that the high levels of MDA may increase the level of ROS and RNS in the host, which plays a key role in NDV infection. The reduction in the concentration of MDA in the brain and bursa of Fabricius of the challenged and vitamin-supplemented group showed that Vitamins E and C are potential immunomodulators and can reduce the effect of NDV-induced oxidative stress in the brain and bursa of Fabricius of chickens.

NO is an important metabolite that plays a key role in the host defense system by recruiting cells across vascular epithelial barriers during infections [35]. However, higher production of NO causes decreased protein function, which leads to tissue and DNA damage and increases disease pathogenicity, whereas a lower production of NO increases the survival rate of viruses, such as the paramyxoviruses [36]. A higher NO concentration was observed in the serum, brain, and bursa of Fabricius of Group 2 and chickens infected with NDV/supplemented with Vitamins E, C, and E + C. However, the NO concentration in the serum, brain, and bursa of Fabricius of the vitamin-supplemented group was lower than that in the nonsupplemented group. This finding is consistent with that of the other studies demonstrating that NDV can induce NO production in peripheral blood mononuclear cells and heterophils [2,37,38]. They suggested that high levels of NO can be attributed to tissue damage in the brain and bursa of Fabricius of chickens and can be a contributing factor to increased mortality associated with ND infection as it increases viral replication. The increased viral replication explains the corresponding higher mortality rate observed in Group 2, but supplementation with either Vitamin E or C reduced the tissue damage in the brain and bursa of Fabricius, which increased the survivability of the chickens compared with Group 5. This finding is inconsistent with that of previous studies on the combined supplementation of Vitamins E and C, which showed a strong immune and antioxidant effect compared with a single-dose administration of the vitamins. The differences observed may be due to the route of supplementation; the previous studies supplemented through the interperitoneal route [39] and drinking water [40] were absorption of these vitamins into the body were faster, whereas our study supplemented through feed inclusion and feed digestion may have slowed absorption of the vitamins. Although vitamin supplementation through feed inclusion was not very effective in our study, the inclusion of drugs in feed is a common and practical method of drug administration for poultry.

GSH is a major cellular non-enzymatic antioxidant that plays a role in detoxifying different metabolites and maintaining the redox balance [41]. SOD is an important enzyme that provides protection against lipid peroxidation [42], whereas CAT is involved in the breakdown of hydrogen peroxide. Decreases in GSH concentration and activities of CAT and SOD were observed in Group 2 and the NDV-infected/Vitamin E-, Vitamin C-, and Vitamin E + C-supplemented groups. This finding is consistent with that of [2], indicating that Vitamin E supplementation resulted in increased activity of antioxidants. The decreases observed in the concentration of GSH and enzymatic activities of SOD and CAT were more significant in Groups 2 and 5 than in Groups 3 and 4, indicating that the individual supplementation of Vitamin E or C partially prevented oxidative stress by maintaining the levels of the cellular antioxidants in the blood and tissues.

Increases were observed in the antibody titer in Group 2 and the NDV-challenged and vitamin-supplemented chickens. However, the individual supplementation of either Vitamin C or E resulted in a stronger immune response. Rehman *et al*. [2], Rehman *et al*. [14] also suggested that vitamin supplementation may not reduce viral replication, but due to the immunomodulatory effect of Vitamin E or C, the viral load may be reduced and the immune response may be maintained.

No significant (p>0.05) reduction was observed in the erythrocytic parameters in the NDV-challenged group, which could be due to the acute nature of infection induced by the velogenic KUDU 113 virus and the ability of vaccination and vitamin supplementation to prevent the destruction of the RBC. This was similarly observed by [43,44]. Furthermore, the nonsignificant increase in the total WBC is usually due to heterophilia and shows the severity of the inflammatory process in NDV infections [44].

## Conclusion

The results of this study suggest that the Nigerian velogenic strain of NDV (KUDU 113), which is known to cause devastating outbreaks in chickens, causes oxidative stress in the plasma, brain, and bursa of Fabricius by increasing the levels of MDA and NO, reducing the levels of GSH, and decreasing the activities of SOD and CAT. Furthermore, the individual supplementation with Vitamin E or C is found to be more effective in ameliorating oxidative stress, improving the immune response, and reducing mortality in KUDU 113 infections when compared with the combined supplementation of Vitamins C and E.

## Authors’ Contributions

WSE, BA, and ONO: Conceived and designed the experiment. TMO, NHI, JNO and IJU: Performed the experiments. TMO: Analyzed the data. OON: Wrote and revised the manuscript. All authors read and approved the final manuscript.
